# Supplementary figures and images for: Integrated DNA Copy Number and Expression Profiling Identifies IGF1R as a Prognostic Biomarker in Pediatric Osteosarcoma
Source: Int J Mol Sci. 2022 Jul 21;23(14):8036. doi: 10.3390/ijms23148036 (PMC9319262; doi:10.3390/ijms23148036)

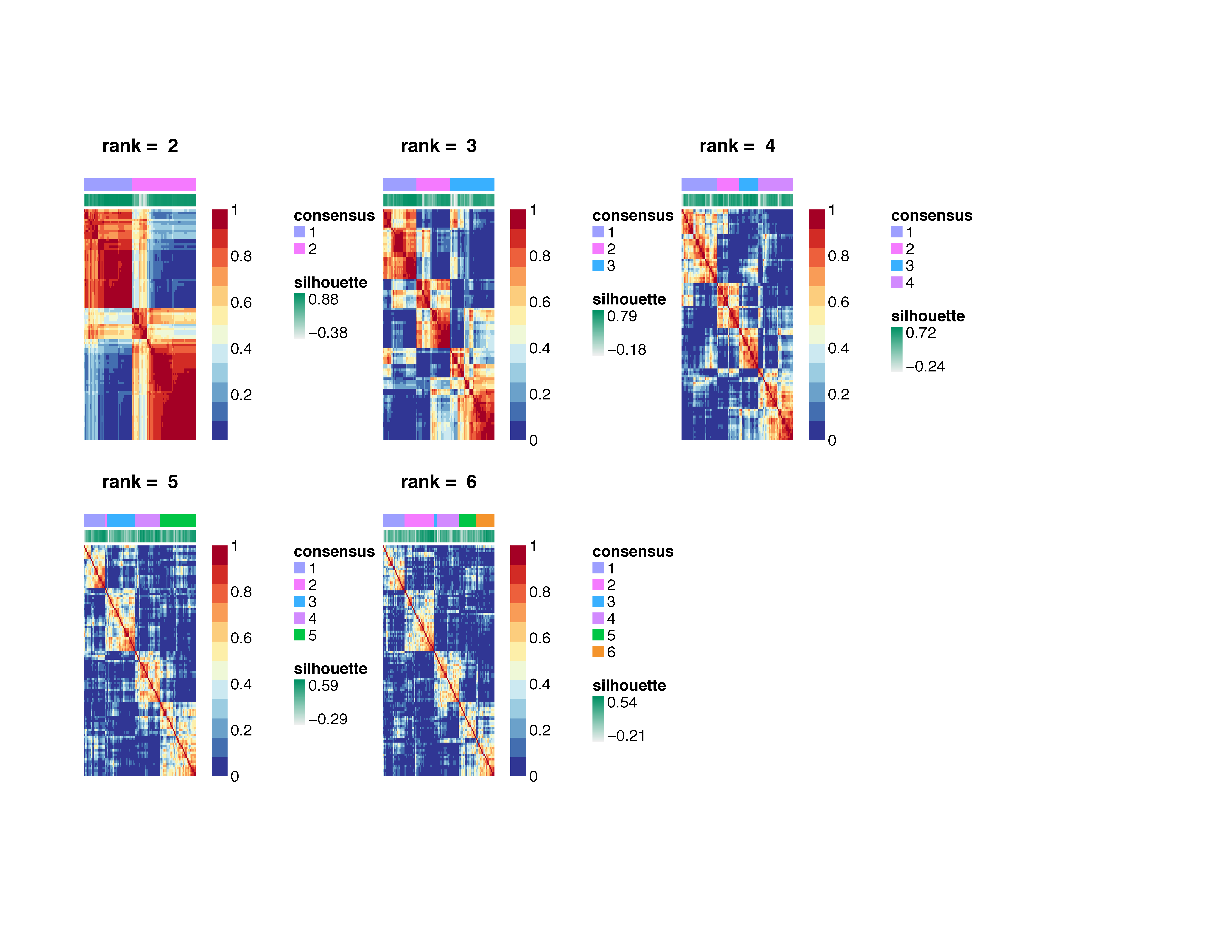

Supplement: Supplementary file 1 [file ijms-23-08036-s001.zip › Supplementary.Figures.Tables/Supplementary Figures/Figure S1.tif]

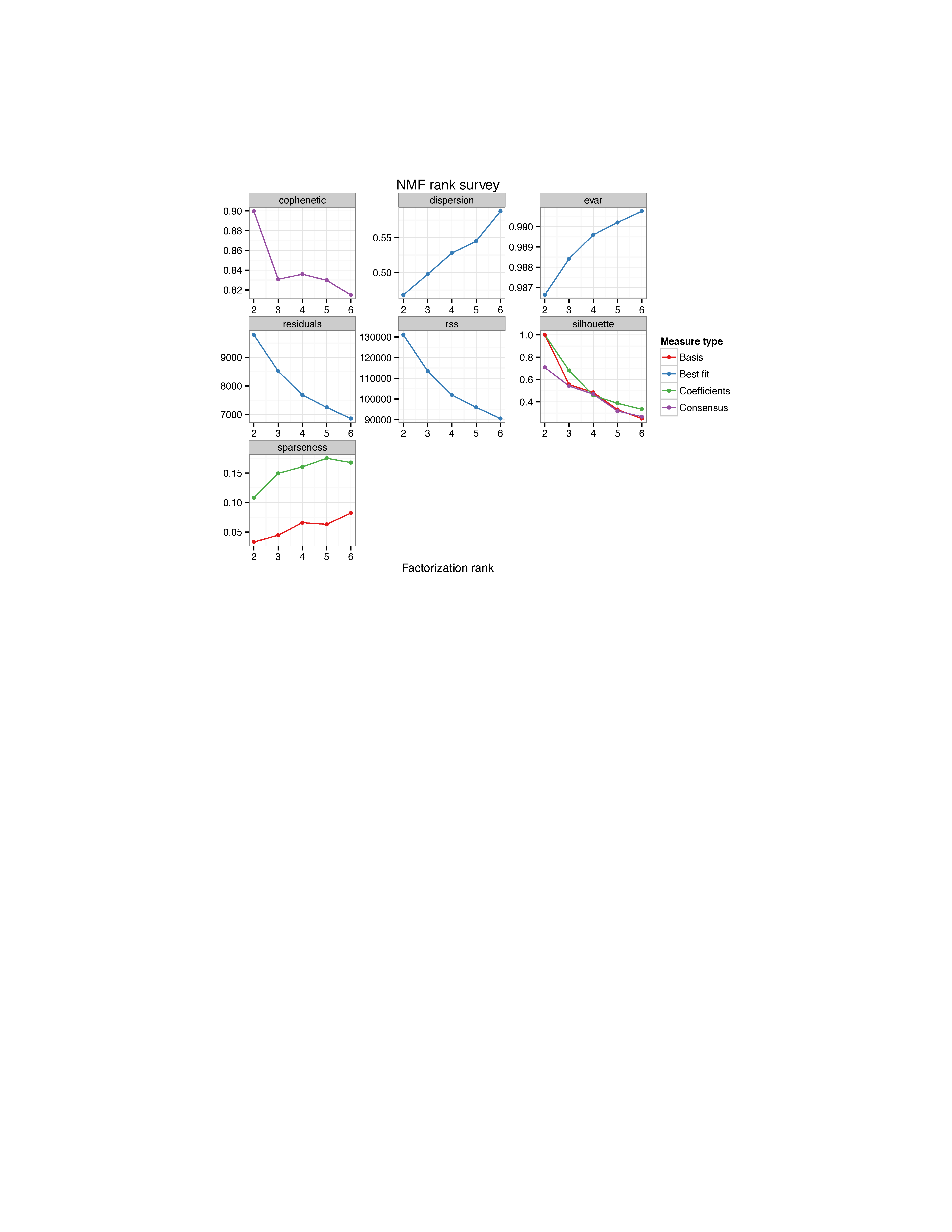

Supplement: Supplementary file 1 [file ijms-23-08036-s001.zip › Supplementary.Figures.Tables/Supplementary Figures/Figure S2.tif]

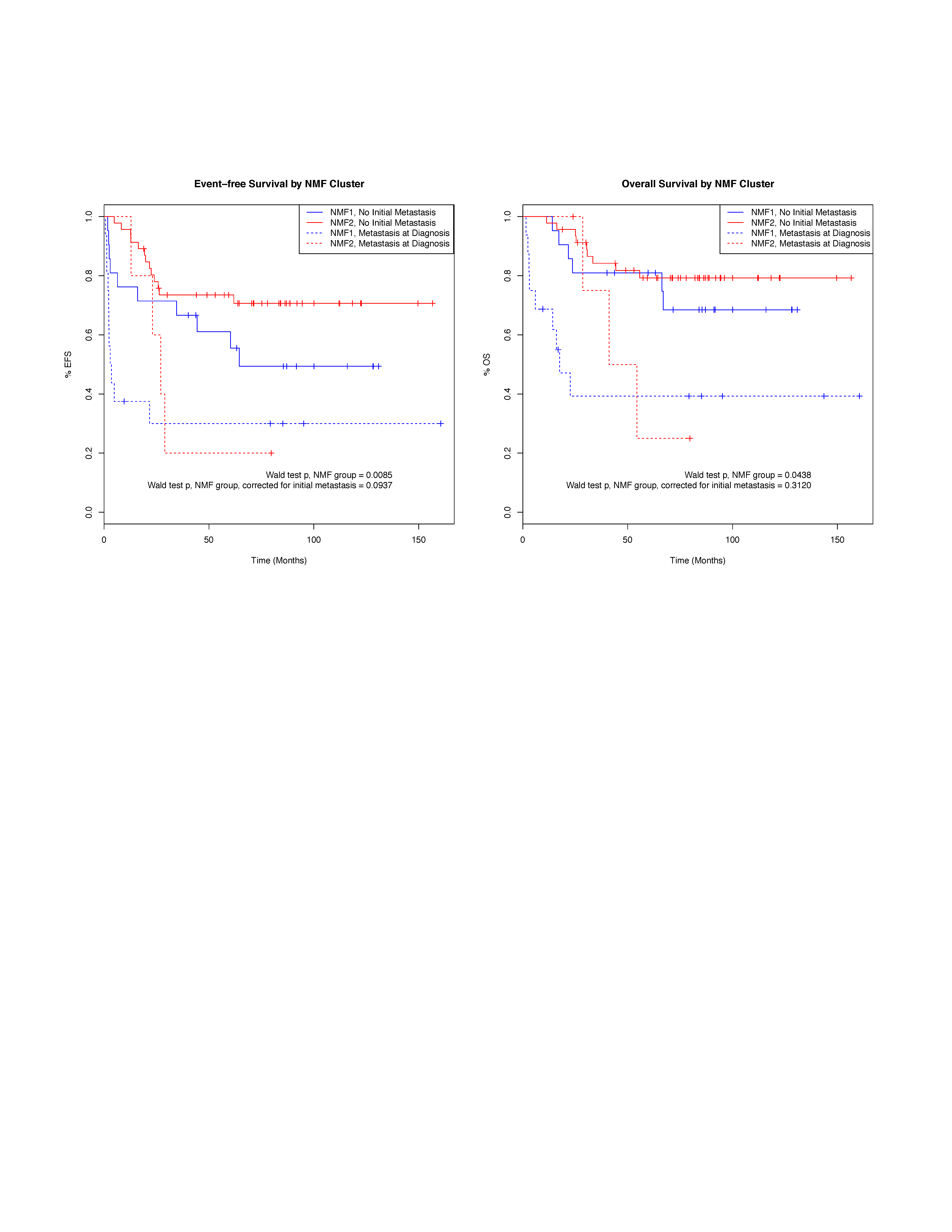

Supplement: Supplementary file 1 [file ijms-23-08036-s001.zip › Supplementary.Figures.Tables/Supplementary Figures/Figure S3.tif]

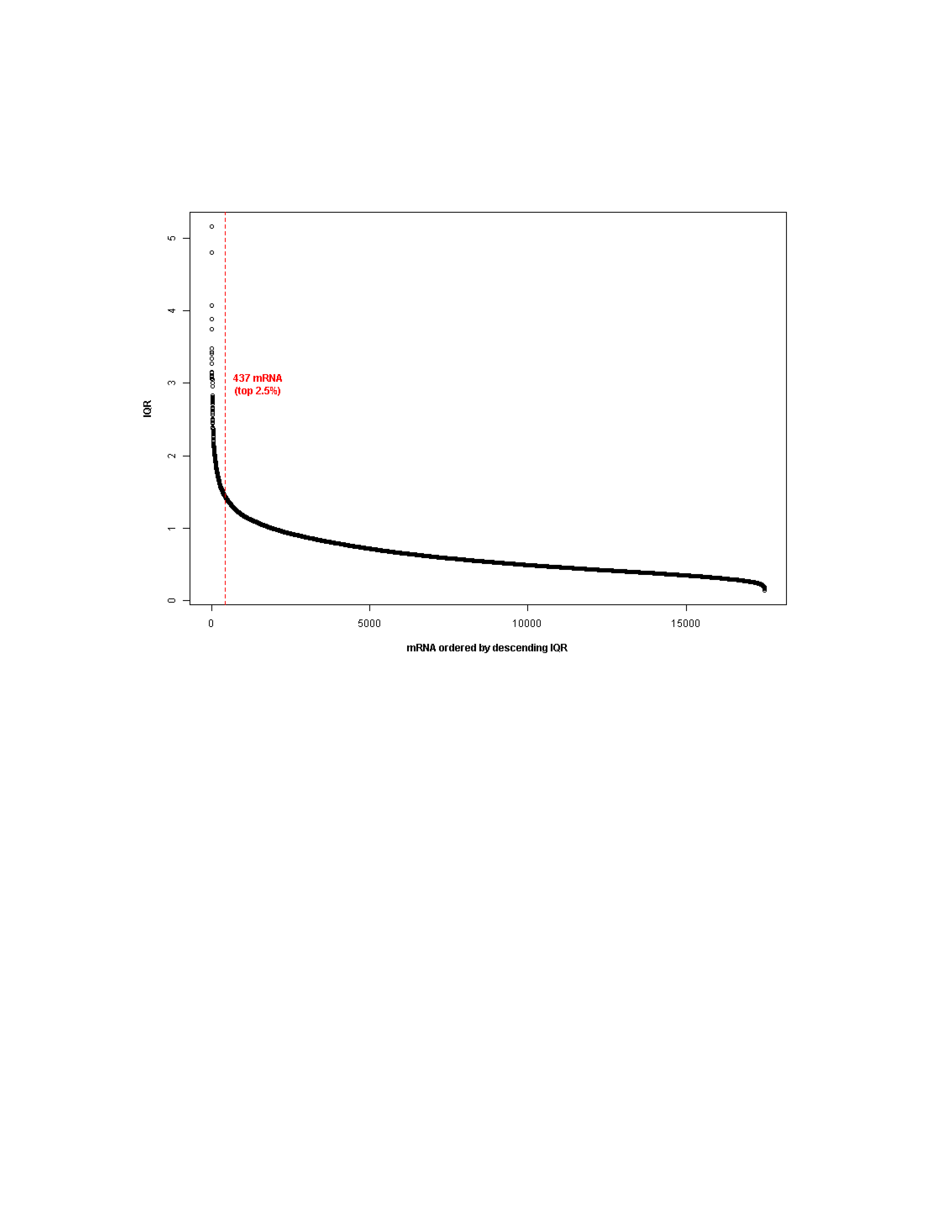

Supplement: Supplementary file 1 [file ijms-23-08036-s001.zip › Supplementary.Figures.Tables/Supplementary Figures/Figure S4.tif]

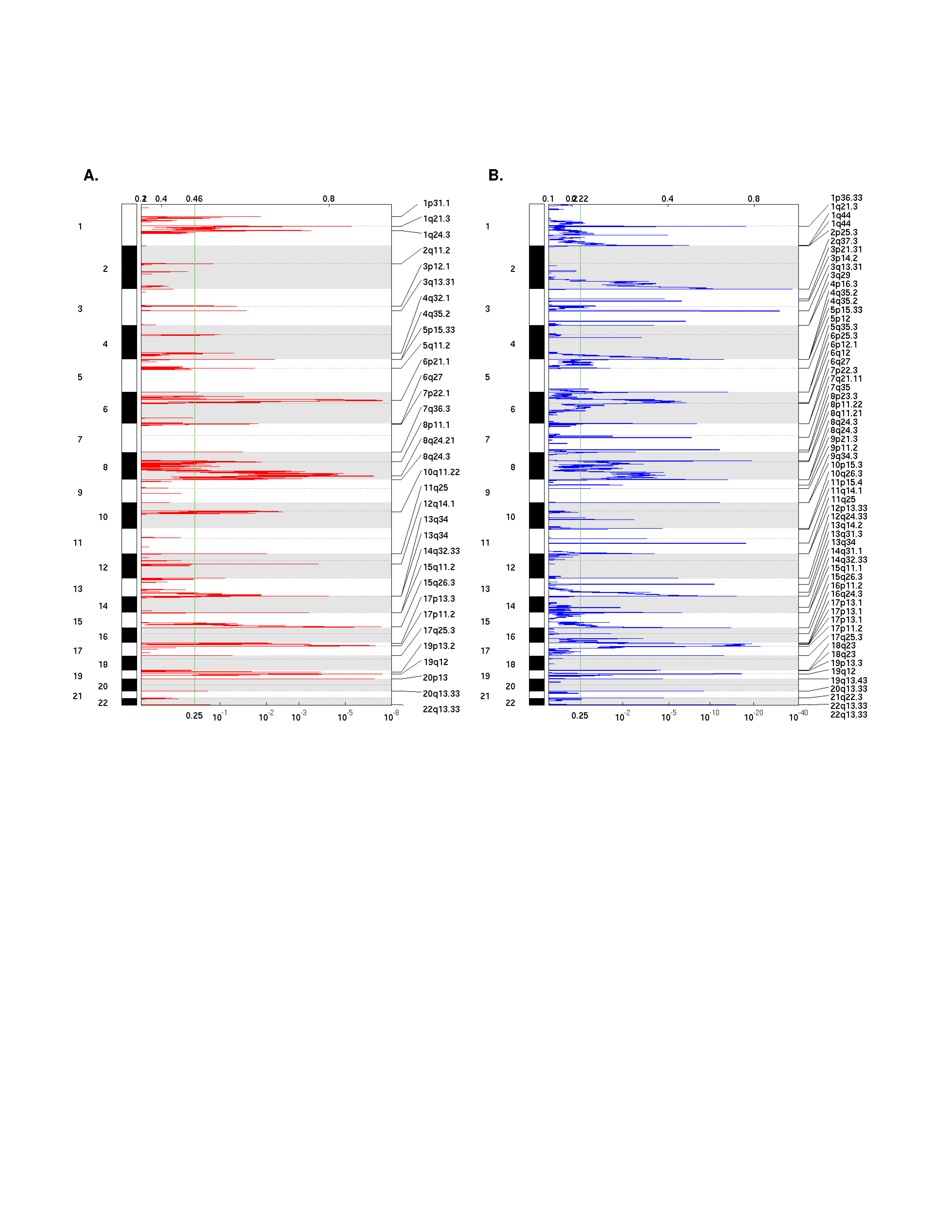

Supplement: Supplementary file 1 [file ijms-23-08036-s001.zip › Supplementary.Figures.Tables/Supplementary Figures/Figure S5.tif]

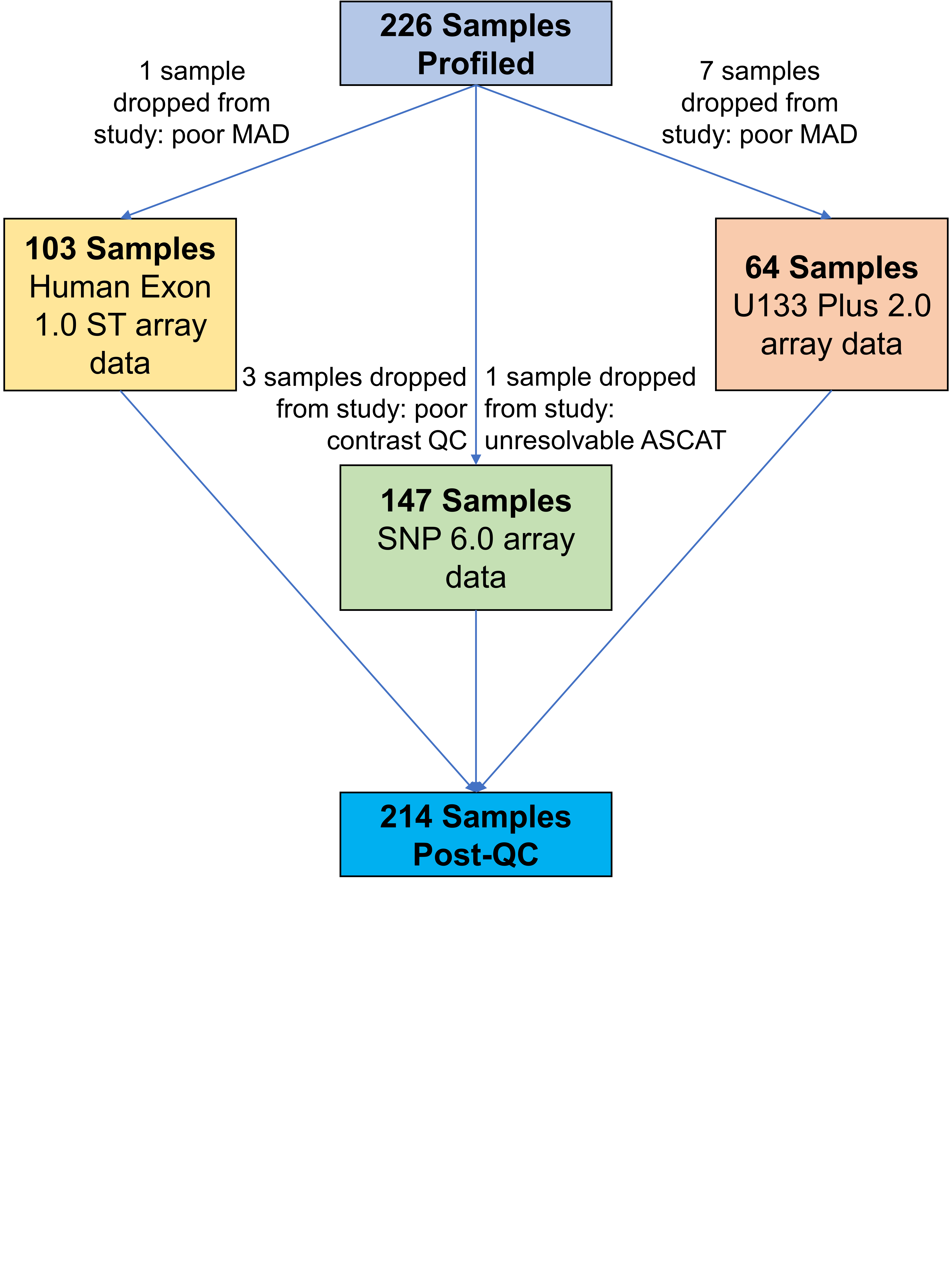

Supplement: Supplementary file 1 [file ijms-23-08036-s001.zip › Supplementary.Figures.Tables/Supplementary Figures/Figure S6.tif]
